# Complete Genome Sequence of Escherichia coli Phage Pisces

**DOI:** 10.1128/MRA.01054-19

**Published:** 2019-09-26

**Authors:** Haydee Diaz, Kristin Graham, Russell Moreland, Mei Liu, Jolene Ramsey

**Affiliations:** a Center for Phage Technology, Texas A&M University, College Station, Texas, USA; Loyola University Chicago

## Abstract

Podophage Pisces was isolated against Escherichia coli strain 4s from wastewater samples. Pisces is a T7-like phage, and all 49 predicted protein-coding genes in it are present on a single strand and are surrounded by 190-bp terminal repeats. Due to its similarity to other T7-like phages, 61% of Pisces genes were assigned a predicted function.

## ANNOUNCEMENT

Escherichia coli, a Gram-negative bacillus and nonsporulating facultative anaerobe, is found in the gut microbiota and feces of many warm-blooded animals and reptiles (1). While E. coli is a commensal microorganism, it can turn into an opportunistic pathogen, and further knowledge about bacteriophages that affect commensals is needed. Here, the isolation and genome sequencing of podophage Pisces, which infects E. coli 4s, are reported.

Phage Pisces was isolated on lawns of E. coli 4s grown aerobically in lysogeny broth and agar at 37°C from filtered (0.2-μm-pore-size) wastewater treatment plant samples in College Station, TX, by the soft-agar overlay method (2, 3). Pisces was negatively stained with 2% (wt/vol) uranyl acetate and viewed by transmission electron microscopy at the Texas A&M Microscopy and Imaging Center (4). Pisces genomic DNA was purified by the previously described shotgun library prep modification to the Promega Wizard DNA clean-up system (5). Sequencing libraries were prepared using an Illumina TruSeq Nano low-throughput kit and sequenced on an Illumina MiSeq platform with paired-end 250-bp reads using v2 500-cycle chemistry. All analyses were done using annotation tools hosted in the Center for Phage Technology Galaxy and Web Apollo instances (https://cpt.tamu.edu/galaxy-pub) (6, 7). The 565,076 sequence reads from the index containing the phage genome were quality controlled with FastQC (https://www.bioinformatics.babraham.ac.uk/projects/fastqc/) and then trimmed with the FASTX-Toolkit v0.0.14 (http://hannonlab.cshl.edu/fastx_toolkit/). The Pisces genome was then assembled via SPAdes v3.5.0 into a single contig with 184.9-fold coverage (8). The raw contig sequence was verified by matching Sanger sequencing of a PCR product amplified from the genomic DNA using primers designed across the ends of the contig (forward primer 5′-CATTATGGCTGACCCTCAGTTC-3′ and reverse primer 5′-AAGTCCGGCCCAGTAGATTA-3′). Protein-coding genes were predicted using GLIMMER v3.0 and MetaGeneAnnotator v1.0 (9, 10). A lack of tRNAs was confirmed using ARAGORN v2.36 (11). Functions of protein-coding genes were predicted using InterProScan v5.33-72, BLAST v2.2.31 with a 0.001 maximum expectation value, TMHMM v2.0, and LipoP v1.0 at default settings (12–15). A BLAST comparison was performed against NCBI nonredundant and UniProtKB Swiss-Prot and TrEMBL databases (16). Genome wide sequence similarity was calculated by progressiveMauve v2.4.0 (17). Rho-independent termination sites were annotated from TransTermHP v2.09 (18). Unless otherwise stated, all tools were executed using default parameters.

Pisces has a 39,497-bp genome of 53.2% G+C content with 49 predicted protein-coding genes on a single strand at 92.1% coding density. Compared to other phages, the closest relative to Pisces with 83.88% nucleotide identity is Escherichia phage IMM-002 (GenBank accession no. MF630921), where 40 proteins are similar. Phage IMM-002 and its relatives are all T7-like phages. The Pisces genome was reopened at short direct terminal repeats of 190 bp predicted by PhageTerm (19), as expected for a T7-like phage.

Due to its similarity to the heavily studied phage T7 (GenBank accession no. NC_001604), 30 of the protein-coding genes of Pisces were assigned a predicted function. The suite of internal virion proteins, including the peptidoglycan transglycosylase (NCBI accession no. QEG09596), were found. The following lysis genes are found separately throughout the genome: endolysin amidase (NCBI accession no. QEG09573), holin class II (NCBI accession no. QEG09598), and embedded o-spanin/i-spanin (NCBI accession no. QEG09601 and QEG09600, respectively).

### Data availability.

The genome sequence and associated data for phage Pisces were deposited under GenBank accession no. MK903277, BioProject accession no. PRJNA222858, SRA accession no. SRR8893603, and BioSample accession no. SAMN11414488.
